# Biomechanical and clinical relationships between lower back pain and knee osteoarthritis: a systematic review

**DOI:** 10.1186/s13643-022-02164-3

**Published:** 2023-03-02

**Authors:** Piyumi Amarasinghe, Surangika Wadugodapitiya, Ishanka Weerasekara

**Affiliations:** 1 Department of Physiotherapy, Faculty of Allied Health Sciences, University of Peradeniya, Peradeniya, Sri Lanka; 2 District General Hospital, Embilipitiya, Sri Lanka; 3 Department of Health and Functioning, Faculty of Health and Social Sciences, Western Norway University of Applied Sciences, Bergen, Norway; 4 School of Health Sciences, The University of Newcastle, Callaghan, NSW Australia; 5 Faculty of Health and Medical Sciences, School of Allied Health Science and Practice, The University of Adelaide, Adelaide, SA Australia

**Keywords:** Knee osteoarthritis, Low back pain, Spinal alignment, Biomechanics, Mechanical back pain, Lumbar radiculopathy, Back pain, Knee pain

## Abstract

**Background:**

Osteoarthritis (OA) and lower back pain (LBP) are most common health problems which lead to pain and disability. This study aimed to systematically review the evidence to find any relationship between knee osteoarthritis (KOA) and LBP or any potential causation.

**Methods:**

The databases of Scopus, MEDLINE, and Embase were searched from inception to 01 October 2022. Any study published in English assessing live humans over 18 years with KOA and LBP was eligible to be included. Studies were independently screened by two researchers. Data of the included studies were extracted based on the participants, outcomes related to knee and lumbar spine, reported association or causation between LBP and KOA, and study design. Data were narratively analyzed and presented as graphs and table. Methodology quality was assessed.

**Results:**

Of 9953 titles and abstracts, duplicates were removed, and 7552 were screened. Altogether, 88 full texts were screened, and 13 were eligible for the final inclusion. There were some biomechanical and clinical causations were observed for the concurrent presence of LBP and KOA. Biomechanically, high pelvic incidence is a risk factor for development of spondylolisthesis and KOA. Clinically, knee pain intensity was higher in KOA when presents with LBP. Less than 20% of studies have justified their sample size during the quality assessment.

**Discussion:**

Development and progression of KOA in patients with degenerative spondylolisthesis may be induced by significantly greater mismatches of lumbo-pelvic sagittal alignment. Elderly patients with degenerative lumbar spondylolisthesis and severe KOA reported a different pelvic morphology, increased sagittal malalignment with a lack of lumbar lordosis due to double-level listhesis, and greater knee flexion contracture than in patients with no to mild and moderate KOA. People with concurrent LBP and KOA have reported poor function with more disability. Both LBP and lumbar kyphosis indicate functional disability and knee symptoms in patients with KOA.

**Conclusions:**

Different biomechanical and clinical causations were revealed for the concurrent existence of KOA and LBP. Therefore, careful assessment of both back and knee joints should be considered when treating KOA and vice versa.

**Systematic review registration:**

PROSPERO CRD42022238571

**Supplementary Information:**

The online version contains supplementary material available at 10.1186/s13643-022-02164-3.

## Background

Osteoarthritis (OA) and lower back pain (LBP) are most common health problems worldwide [1]. OA is a major form of arthritis causing pain and disability. Approximately 15% of the world population is affected with OA [2, 3]. Direct lifetime medical expenses for adults in the United States (US) related to knee osteoarthritis (KOA) are reported as US $12,400 [4]. LBP is also very common in western countries, affecting 80% of people at any point in their lifetime [3]. The healthcare cost of it is £1632 million in the USA [5].

Some patients with KOA may not respond well to the recommended knee exercises. This may be due to the possibility of additional concurrent conditions affecting KOA, such as LBP. LBP is a common complaint in individuals with KOA [6, 7], and concurrent LBP was reported as 57.4% of patients with KOA [7]. The quality of life (QOL) is affected in people with concurrent OA and LBP [1]. Some evidence was found in literature to support the association between LBP and KOA and causations. Any structural or functional factors that could cause concurrent LBP in KOA, such as increased BMI or repetitive posture, should be carefully taken into account when managing KOA. The mechanism, nature, and cause of any common factors leading to concurrent LBP in KOA are still unclear, and to date, no systematic review has been conducted to pool this data. Therefore, it is worthwhile to collate evidence on concurrent existence of LBP in KOA, aiming to investigate any relationship and causations between these two conditions.

It was reported that there was a higher prevalence (57.4%) of the coexistence of KOA and LBP [7] and the higher knee pain intensity in KOA when presented with LBP [8]. These two conditions might be biomechanically related [9, 10]. There is a paucity of pooled information in the literature, and inconsistent findings have been reported about the alignment of the spine, the pelvis, the lower extremities, and associated musculature attached to those structures [11] in relation to the concurrent existence of these two debilitating conditions [12].

There is no previous systematic review to date, assessing the relationships or causation for this concurrent existence of KOA and LBP. Therefore, the objective of this systematic review is to explore whether there is any relationship or potential causation for the concurrent existence of these two conditions.

## Method

### Registration

The protocol for this systematic review was registered with the International Prospective Register of Systematic Reviews (PROSPERO) (CRD42022238571) on 14 January 2022.

### Aims

The objective of this study is to explore whether there is any relationship or potential causation for the concurrent existence of LBP and KOA.

### Design

This systematic review was conducted and guided by the Preferred Reporting Items for Systematic Reviews and Meta-Analyses (PRISMA) guidelines [13] (Additional file 1).

### Search strategy

A search of electronic databases including MEDLINE, Embase, and Scopus were conducted from inception to 22 February 2021. This search was updated on 01 October 2022. A search strategy was developed for the main search strings of “knee osteoarthritis” and of “low back pain.” Keywords for “knee osteoarthritis” were degenerative joint disease of the knee, degenerative arthritis of knee, and osteoarthritis of the knee. Key words for “low back pain” were low back ache, sacroiliac joint pain, mechanical back pain, and lumbar radiculopathy. These terms were utilized alone and in combinations during the search. The search strategies are available in Additional file 2. For this review, KOA was described as progressive destruction of articular cartilage and a disease involving whole knee joint [13], while LBP was described as pain involving or derived from structures in the lumbosacral region between the lower posterior margin of the rib cage and the horizontal gluteal fold [14].

### Identification and selection of studies

Below inclusion and exclusion criteria were applied in deciding the eligibility of the studies.

#### Inclusion criteria

- Any study assessing live humans over 18 years with KOA and back pain
- Any study comparing the condition to their non-affected lower limb or to healthy people
- Any variable assessing biomechanical (structural outcomes such as angles, alignments, range of motion (ROM)) or clinical outcomes (function/disability, pain) of lumbar area of KOA
- Any study design except case studies, case series published in peer-reviewed journals
- Studies in English

#### Exclusion criteria

- Studies on children, animals, or cadaveric studies
- Studies with other arthritic conditions or joints other than knee joint
- Conference abstracts
- Nonoriginal study designs such as commentaries, research notes, editorials, or letters
- Any form of reviews

Data search was exported to EndNote reference manager software (EndNote version 9.3.3., Clarivate, Philadelphia, USA) and then to the Covidence systematic review management software (Covidence systematic review software, Veritas Health Innovation, Melbourne, Australia) to remove duplicates and for screening. Screening was carried out independently by two researchers: title and abstracts first and then full texts. Discrepancies were resolved by consensus, and any conflicts were resolved by a third researcher. Descriptive data were extracted by the first author, using an extraction table in Microsoft Excel. Authors were contacted when there is missing or no sufficient details during data extraction from the eligible studies. The following details were extracted: publication details, participant characteristics, details about the conditions, details of the comparator, and outcome measures (any existing association between two conditions).

### Data analysis

Narrative synthesis was carried out and presented graphically and as tables as appropriate. No meta-analysis was carried out since there was a lack of studies assessing the same outcomes and due to a lack of meaningful data to pool together.

### Assessment of methodological quality

The methodological quality of the individual studies was assessed by the first author using the quality assessment tool for observational cohort and cross-sectional studies [15]. This has 14 items assessing the quality of the methods on research question, study population, eligibility criteria, sample size, outcome measurements, timeframe, exposure, follow-up, and analysis. Any clarifications were discussed with the research team when required.

## Results

### Selection and characteristics of included studies

**Fig. 1:**
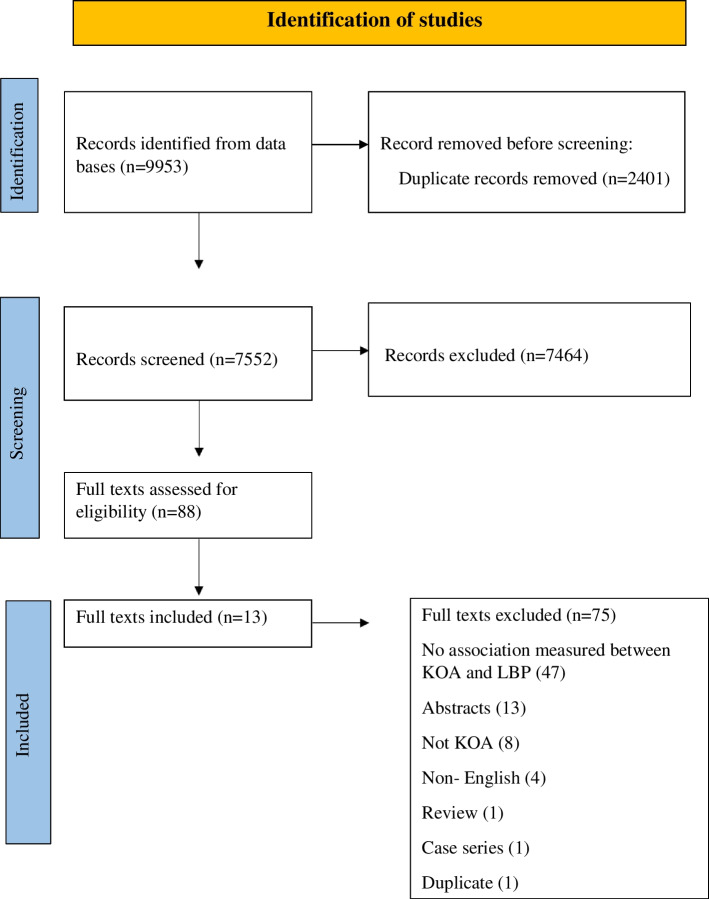
Flow chart of the study

The database search identified 7552 studies after removal of 2401 duplicates. Following the first stage of screening (title and abstract), 88 full texts were screened to identify eligible studies for final inclusion. A further 75 studies were excluded at the second stage of screening (full text), mainly because the studies were not assessing any causation or relationship, not both the interested conditions were explored, or because of non-peer-reviewed publications such as conference proceedings, commentaries, and research notes. Thirteen studies [6–9, 12, 16–23] were therefore included into the final analysis of the current review (Table 1) (Fig. 1).

**Table 1:** Description of eligible studies

Study	Design	Sample (sample size (number of males), laterality, knee condition)	Age (mean ± SD)	BMI (kg/m^2^) (mean ± SD)	Prevalence of reported LBP	Outcomes related to knee	Outcomes related to lumbar spine	Causation/relationships
Chang et al., 2014 [17]	Retrospective study	225 (15 M) Laterality — NS Preoperative primary TKA due to advanced primary KOA	69 + 6.5	26.8 + 3.5	100%	Pain, stiffness, physical function (WOMAC) Physical status (SF-36)	Pain VAS Radiographical changes	Diminished pain (*β*, 0.08, *p* = 0.28), physical function (*β*, −0.02, *p* = 0.80), and physical status (*β*, −0.001, *p* = 0.99): not associated with radiographic severity of lumbar spine degeneration LBP severe grade (*VAS* 7–10): associated with knee pain (regression coefficient with 95% *CI* −11.66 (−21.50 to −1.82)) Function: Affected by severe LBP (VAS scores 7–10) (regression coefficient with 95% *CI* −17.8 (−26.36 to −9.24)) Poorer function: Associated with moderate symptom grade (regression coefficient with 95% *CI* −5.64 (−12.24 to 1.07) Physical status: Affected by severe LBP (VAS scores 7–10) (regression coefficient with 95% *CI* −1.61 (−4.74 to −1.52) Radiographic lumbar spine degeneration: Found in all study subjects without exception (patients’ percentage below mentioned) • Mild degeneration: 11% • Moderate degeneration: 72% • Severe degeneration: 17% Lumbar spine symptoms LBP VAS scores (mean ± SD, 3.1 ± 2.7): Considerable proportion of patients had coexisting moderate to severe symptoms at the time of TKA (patients’ percentage below mentioned) • No/mild pain VAS 0–3 (60%) • Moderate pain VAS 4–6 (28%) • Severe pain VAS 7–10 (12%)
Huang et al., 2014 [18]	RCT	Eight — severe bilateral KOA patients with chronic nonspecific LBP group Seven —healthy participants aged 23.00 (20.00/24.00) years, without OA, LBP, or other musculoskeletal symptoms (control group), 8 — bilateral KOA patients without LBP (NLBP group)	NS	NS	NS	Knee flexion Pain Functional disability of the patients with knee OA (Lequesne’s index scores)	Trunk flexion Pelvic anterior tilt Anterior trunk inclination angle Physical disability due to back pain (RDQ) Back pain intensity (ODI)	Trunk flexion angles (°): Smaller in KOA patients compared to healthy people without KOA or LBP (median (IQR), with LBP −27.65 (−33.07/−20.10); non-LBP −27.44 (−32.83/−24.30); healthy −40.43 (−46.46/−36.44)) Trunk rotation angle (°): Smaller in NLBP group than that of the controls (median (IQR), with NLBP 6.01 (3.89/8.23); controls 9.15 (6.57/10.25) Knee flexion angles in ipsilateral side of bending (°): Significantly smaller when doing the downward pickup movement in both the LBP and NLBP groups (median (IQR), with LBP −7.54 (−12.31/−3.78); non-LBP −6.39 (−12.95/−4.05); controls −19.89 (−31.63/−6.50)) Pelvic anterior tilt (°): Greater in KOA compared to the healthy people (median (IQR), with LBP −44.68 (−50.18/−40.52); NLBP −45.83 (−48.56/−39.38); healthy −32.61 (−37.05/−28.47)) Anterior trunk inclination angle(°): No significant difference between KOA and healthy people (median (IQR), with LBP −82.13 (−89.33/−73.23); NLBP −83.96 (−88.80/−74.07); controls −85.05 (−85.96/81.92) Physical disability: Higher in LBP group (median (IQR), with LBP 9 (7.3/10.8); non-LBP 3.5 (2.0/5.8)) Levels of back pain intensity component: Higher in LBP group (median (IQR), with LBP 1.0 (1.0/2.0); non-LBP 0.5 (0.0/1.0)) Pain and functional disability of the patients with knee OA: No statistically significant difference between LBP and non LBP (median (IQR), with LBP 11.0 (9.3/15.0); 13.0 (10.0/14.0))
Iijima et al., 2018 [8]	Cross-sectional study	260 (22.3% M) — community-dwelling participants with knee OA (K/L grade ≥ 1), OA with LBP −151 and OA without LBP −109	OA with LBP 68.6 ± 9.3, OA without LBP 70.7 ± 9.0	OA with LBP 22.9 ± 3.7, OA without LBP 22.0 ± 3.2	58.1%	Knee pain severity and disability JKOM	Pain NRS	LBP: Associated with increased disability level (*β*: 0.69; 95% *CI*: 0.01 to 1.37) (*p* = 0.05) Relationships of LBP and disability level: Slightly increased in moderate to severe LBP (*β*:1.01; 95% *CI*: 0.22 to 1.80) (*p* = 0.01) Relationship between knee pain intensity and disability level: Higher in individuals with LBP (*β*: 0.62; 95% *CI*: 0.51 to 0.73) than in those without LBP (*β*: 0.40; 95% *CI*: 0.32 to 0.49)
Iwamura et al., 2020 [19]	NS	57 (10 M) DS patients who complicate KOA (KOA group), 127 (33 M) DS patients without KOA (non-KOA group)	72.7 ± 7.0, 69.4 ± 8.2	24.5 ± 3.8, 22.8 ± 2.8	NS	NS	Parameters in lumbo-pelvic sagittal alignment: PI, PT, LL, PI-LL, SS	Lumbo-pelvic sagittal alignment: Development and progression of KOA in DS patients is induced by significantly greater (*p* = 0.02) mismatches of lumbo-pelvic sagittal alignment Parameters in lumbo-pelvic sagittal alignment: PI (°); PT(°); LL(°); PI-LL and SS (°) of KOA group and non-KOA group were mean ± SD, 27.2 ± 9.8 and 22.2 ± 8.6, 40.4 ± 15.8 and 42.6 ± 14.3, 17.9 ± 15.1 and 10.3 ± 12.9, and 30.6 ± 10.0 and 30.6 ± 8.9, respectively Significant difference was observed in the rate of double adjacent level spondylolisthesis (*p* = 0.02) and in the following sagittal parameters: PT (*p* < 0.01), PI-LL (*p* < 0.01)
Kohno et al., 2020 [20]	Retrospective study	Patients with DLS comorbid with 42-mild OA group, 28 — moderate OA group, 40 — severe OA group	74	22.6 ± 3.2 23.4 ± 3.2 23.9 ± 2.9	NS	KFA	PI PT	PI, PT, and KFA: Significantly greater in severe OA group, than mild OA group along with a smaller degree of LL than the mild-OA group preoperatively (all *p* < 0.05) PI (°): Significantly greater in severe OA group ( mean ± SD, 7 ± 8.7) than the mild OA group (51.8 ± 9.6) (*p* = 0.05) PT (°): Significantly greater in severe OA group ( mean ± SD, 28.8 ± 9.3) than the mild OA group (20.1 ± 8.3) (*p* < 0.01) LL (°): Significantly smaller in severe OA ( mean ± SD, 38.7 ± 12.2) than the mild OA (45.6 ± 13.0) (*p* = 0.04) KFA (°): Significantly greater in severe OA ( mean ± SD, 10.1 ± 5.3) group than the mild-OA (4.9 ± 6.8) group preoperatively (*p* = 0.02) Rate of radiographic ASD: Higher in the severe-OA group than in the mild-OA group (*p* = 0.02) patients percentage (38%) PT (°): Significantly greater in patients with ASD ( mean ± SD, 26.2 ± 7.0) in the severe-OA group than the patients without ASD (34.1 ± 10.8) (*p* = 0.02) LL (°): Less in patients with ASD (mean ± SD, 34.9 ± 14.6), than without ASD (40.6 ± 9.9) (*p* = 0.26) Rate of double-level listhesis: Significantly higher in the severe-OA group compared with the other groups (*p* = 0.01) (patients number %), mild OA group 12; moderate OA 31; severe OA 40
Staibano et al., 2014 [21]	Prospective cohort study	491 (40.1% M) patients with end-stage KOA	67.6 ± 9.6	31.9 ± 6.4	47.3%	NS	Back pain Degree of disability due to back pain (ODI)	Degree of disability due to back pain: Minimal (mean ± SD, 14.5 ±14) due to back pain in preoperative TKA patients with none or very mild LBP (*p* = 0.01) Pain on the ODI: Significantly higher among knee patients with a 68.4% (95% *CI*, 57.4–77.6%)
Stupar et al., 2010 [12]	Population-based cohort study	406 LBP	NS	NS	58%	Pain, stiffness, and physical function (WOMAC)	NS	Pain and disability: Not associated with LBP in individuals with KOA (*β* = 0; 95% *CI*, −3.39 to 3.39; *p* = 0.99)
Suri et al., 2010 [7]	NR	1389 (40.1% M) people with KOA	61.4 ± 9.1	30.2 ± 4.9	57.4%	Pain component (WOMAC)	NS	LBP: Significantly associated with increased functional score (*β* = 1; *p* < 0.01) (WOMAC score with LBP mean ± SD, 6.5 ± 4.1, without LBP 5.2 ± 3.4)
Taniguchi et al., 2021 [23]	Cross-sectional	586 (116 M) participants with x-ray-confirmed KOA	68.8 ± 5.2	NS	NS	Functional abilities related to knee (KSS)	Lumbar kyphosis	LBP and lumbar kyphosis: Independently associated with a lower function (LBP alone MD 95% *CI*, −4.96 (−7.56 to 13, −2.36); lumbar kyphosis alone, −4.47 (−8.51 to −0.43) Coexistence of LBP and lumbar kyphosis −13.86 (−18.86 to −8.86)) Coexistence of LBP and lumbar kyphosis: Was associated with a lower function in women (MD 95% *CI* −4.49 (−6.42 to −2.55))
Van Erp et al., 2020 [16]	Cohort study	421 (116 M) hip and KOA	56.1 ± 5	26.6 ± 4	NS	NS	PI	PI (°): Significantly higher incidence of knee OA was observed in patients with a high PI, compared with those with normal PI (*OR* 1.70, 95% *CI* 1.07 to 2.71) (*p* = 0.02) or low PI (*OR* 1.62, 95% *CI* 1.04 to 2.53 (*p* = 0.03) High PI (> 60°): Is a risk factor for development of spondylolisthesis (L4 to L5, *p* = 0.02) and KOA (*p* = 0.03)
Wang et al., 2016 [9]	Cross-sectional study	59 (16) patients with severe KOA, 58 (14) asymptomatic persons free from KOA	65.9, 62.9	NS	66.1%	NS	Sagittal alignment of the pelvis and hip: PI, PT, SS, PFA, SFA, FI, spinosacral angle, and C7 tilt	Sagittal alignment: No significant difference between KOA patients with and without LBP Comparable PI, SS, and PT values were revealed between the two groups, suggesting similar sagittal morphology and pelvic alignment PI (°): With LBP mean ± SD, (48.5 ± 10.4); without LBP (45.0 ± 10.0) (*p* = 0.68) SS (°): With LBP (36.2 ± 9.2); without LBP (32.9 ± 8.4) (*p* = 0.92) PT (°): With LBP (12.5 ± 6.3); without LBP (12.2 ± 7.1) (*p* = 0.32) Severe KOA patients showed significantly larger FI (11.3° versus 4.2°, *p* < 0.01) and smaller SFA (43.1° versus 51.8°, *p* < 0.01) and PFA (2.2° versus 9.1°, *p* < 0.01) values compared with controls These results indicate flexed knee and hip joints among patients with severe KOA C7 tilt: Significantly smaller among severe KOA patients compared with controls (88.4° versus 92.9°, *p* < 0.01), indicating forward inclination of the spine
Wolfe et al., 1996 [6]	NS	368 (23.1% M) diagnosed clinically as having KOA	NS	31.0	54.6%	NS	Disability (HAQ) Pain VAS	Back pain: Strongly associated with knee pain (*p* = 0.03) Knee pain VAS (1–1.9): *OR* 2.18, 95% *CI* (2.03, 3.83) Knee pain VAS (≥ 2): *OR* 4.89, 95% *CI* (2.60, 9.20) Disability (*p* < 0.01): Strongly associated with back pain (*p* = 0.03) Disability (1–1.9): *OR* 2.12, 95% *CI* (1.37, 3.30) Disability (≥ 2): *OR* 6.84, 95% *CI* (2.87, 16.26)
Yasuda et al., 2020 [22]	Large cohort study of volunteers	396 (160 M) volunteers over 50 years of age	74.4	NS	NS	KL grading scale	Spinopelvic sagittal alignment: PT, PI, LL, thoracic kyphosis, and SVA, ODI	Lumbo-pelvic sagittal alignment: Poor in individuals over 50 years of age with severe KOA and has stronger relationship with progression severity of KOA in women than in men PT (°): Mean ± SD, KL1 (15.8 ± 7.5), KL2 (20.1 ± 8.8), KL3 (21.4 ± 9.2), KL4 (24.7 ± 9.5) (*p* = < 0.01) Degree of disability due to back pain: Progression severity of KOA had more impact on stronger relationship with disability-related LBP in (women > men) (*p* = 0.02) ODI score: KL1 ( mean ± *SD*, 9.9 ± 10.8), KL2 (12.2 ± 11.9), Kl3 (1 ± 12.1), KL4 (16.1 ± 13.0) ODI score: Higher in the KL4 than in the KL1

*Abbreviations*: *ASD* adjacent-segment disease, *BMI* body mass index, *β* beta coefficient, *CI* confidence interval, *DLS* degenerative lumbar spondylolisthesis, *DS* degenerative spondylolisthesis, *FI* femoral inclination, *HAQ* Health Assessment Questionnaire, *JKOM* Japanese Knee Osteoarthritis Measure, *K/L* Kellgren and Lawrence, *KFA* knee flexion angle, *KOA* knee OA, *KSS* knee scoring system, *LBP* low back pain, *LL* lumbar lordosis, *M* male, *MCS* mental component summary, *MD* mean difference, *NS* not specified, *OR* odds ratio, *ODI* Oswestry Disability Index, *OA* osteoarthritis, *PCS* physical component summary, *PFA* pelvic femoral angle, *PI* pelvic incidence, *PI-LL* pelvic incidence-lumbar lordosis, *PT* pelvic tilt, *RDQ* Roland-Morris Disability Questionnaire, *RCT* randomized control study, *ROM* range of motion, *SD* standard deviation, *SFA* sacrofemoral angle, *SS* sacral slope, *SVA* sagittal vertical axis, *SD* standard deviation, *SF*-36 short-form 36, *TKA* total knee arthroplasty, *VAS* visual analogue scale, *WOMAC* Western Ontario and McMaster Universities Osteoarthritis Index

The included studies were conducted in eight countries (Korea, Taiwan, Japan, Canada, Australia, Netherland, Kansas, and China) involving in 4976 participants. Outcome measures assessed were related to the knee were pain, disability, knee range of motion (ROM), and KOA severity grades. There were seven studies assessing biomechanical associations [9, 16, 18–20, 22, 23] and 10 of studies assessing clinical outcomes [6–8, 12, 16–18, 21–23]. There were six studies evaluating the outcomes related to knee pain and disability [7, 8, 17, 18, 20, 23] (Table 2), six studies evaluating the outcomes related to back pain and disability [6, 8, 17, 18, 21, 22], one study measuring the knee flexion angle [20], the spinal ROM [18], and one study evaluating the knee OA severity [22] (Table 2).

**Table 2:** Biomechanical causations/relationships between LBP and knee OA

Study	Biomechanical causations/relationships		
	Spinopelvic alignment	Angles	ROM
Huang et al, 2014 [18]	Pelvic anterior tilt(°): Greater in KOA compared to the healthy people (median (IQR), with LBP −44.68(−50.18/−40.52); NLBP −45.83(−48.56/−39.38); healthy −32.61(−37.05/−28.47))	Anterior trunk inclination angle(°): no significant difference between KOA and healthy people (median(IQR), with LBP −82.13 (−89.33/−73.23); NLBP−83.96(−88.80/−74.07); controls −85.05(−85.96/81.92))	Trunk flexion angles(°): smaller in KOA patients compared to healthy people without KOA or LBP(median (IQR), with LBP −27.65 (−33.07/−20.10) ; non LBP −27.44 (−32.83/−24.30); healthy −40.43 (−46.46/−36.44))
			Trunk rotation angle(°): smaller in NLBP group than that of the controls (median (IQR), with NLBP 6.01 (3.89/8.23); controls 9.15 (6.57/10.25)
			Knee flexion angles in ipsilateral side of bending (°): significantly smaller when doing the downward pick-up movement in both the LBP and NLBP groups (median (IQR), with LBP −7.54 (−12.31/−3.78); non LBP −6.39 (−12.95/−4.05); controls −19.89 (−31.63/−6.50))
Iwamura et al, 2020 [19]	Lumbo-pelvic sagittal alignment:development and progression of KOA in DS patients is induced by significantly greater mismatches of lumbo-pelvic sagittal alignment (*p*=0.02)	PI- significantly greater PI with dominant of double adjacent level spondylolisthesis in patients with concurrent KOA (mean ± SD), (58.0° ± 10.4 ) and DS (p<0.01) than in patients with DS without KOA(52.8° ± 10.0) (*p*<0.01)	Not assessed
	Parameters in lumbo-pelvic sagittal alignment: PT; LL; PI-LL and SS of KOA group and non-KOA group were mean ± SD, 27.2° ± 9.8 and 22.2° ± 8.6; 40.4° ± 15.8 and 42.6° ± 14.3;17.9° ± 15.1 and 10.3° ± 12.9, and 30.6° ± 10.0 and 30.6° ± 8.9, respectively, and significant difference was observed in the rate of double adjacent level spondylolisthesis (*p* = 0.023), and in the following sagittal parameters: PT (*p* < 0.001), and PI-LL (*p* < 0.001)		
Kohno et al, 2020 [20]	PT(°): significantly greater in patients with ASD ( mean ± SD), (26.2 ± 7.0) in the severe-OA group than the patients without ASD (34.1 ± 10.8) (*p*=0.02)	PI, PT, KFA: significantly greater in severe OA group, than mild OA group along with a smaller degree of LL than the mild-OA group preoperatively (all *p* < 0.05)	KFA (°): significantly greater in severe OA (mean ± SD), (10.1 ± 5.3) group than the mild-OA (4.9 ± 6.8) group pre- operatively (*p*=0.02)
	LL(°): less in patients with ASD ( mean ± SD), (34.9 ± 14.6), than without ASD (40.6 ± 9.9) (*p*=0.26)	PI (°): significantly greater in severe OA group (mean ±SD), (7 ± 8.7) than the mild OA group (51.8 ± 9.6) (*p*= 0.05)	
	Rate of double-level listhesis: significantly higher in the severe-OA group compared with the other groups (p=0.01) (patients number %) mild OA group 5 (12); moderate OA 8 (31); severe OA 16 (40)	PT (°): significantly greater in severe OA group (mean ± SD), (28.8 ± 9.3) than the mild OA group (20.1 ± 8.3) (*p* <0.01)	
		LL (°): significantly smaller in severe OA (mean ± SD), (38.7 ± 12.2) than the mild OA (45.6 ± 13.0) (*p*= 0.04)	
Taniguchi et al, 2021 [23]	Lumbar kyphosis: associated with a lower functional abilities with lumbar kyphosis (mean± SD), (77.4 ± 19.1) (p < 0.001) than those without lumbar kyphosis (86.1 ± 15.3) (*p* = 0.03)	Not assessed	Not assessed
Van Erp et al, 2020 [16]	PT (*p*= 0.07) and SS (*p*=0.09): correlated with radiological knee OA KL ≥ 2 and different degree of PI, individuals with high PI had significantly higher scores compared to low PI	PI: high PI (58.3) was associated with higher incidence of knee OA compared to low PI (49.5) (*p* = 0.03)	Not assessed
	Spondylolisthesis were more frequently present in subjects with high PI compared to low PI (L4 to L5; *p* = 0.02 vs L5 to S1; *p* = 0.001)		
	L5 to S1 DDD: occurred more in patients with low PI compared to high PI (*p* = 0.01)		
Wang et al, 2016 [9]	Sagittal alignment: no significant difference between KOA patients with and without LBPComparable PI, SS, and PT values were revealed between the two groups, suggesting similar sagittal morphology and pelvic alignment	Patients with severe KOA showed significantly smaller SFA (43.1° versus 51.8°, *p* < 0.01) and PFA (2.2° versus 9.1°, *p* < 0.01) values compared with controls.	Not assessed.
	PI(°): with LBP (mean ± SD),(48.5±10.4), without LBP (45.0±10.0)(*p*=0.68)	FI: significant backward FI larger FI (11.3° versus 4.2°, *p* < 0.01), hip flexion, and forward spinalinclination in patients with severe KOA compared with asymptomatic persons free from KOA (*p* < 0.01)	
	SS(°): with LBP (36.2±9.2) withoutLBP (32.9±8.4) (*p*=0.92)	FI 10°: showed no significant difference in the prevalence of LBP compared with those with FI > 10° (18/23 versus 21/36 patients, chisquared = 2.5, *p* = 0.11)	
	PT(°):with LBP(12.5±6.3) withoutLBP(12.2±7.1) (*p*=0.32)	C7T: significantly smaller among severe KOA patients compared with controls (88.4° versus 92.9°, *p* <0.001), indicating forward inclination of the spine	
Yasuda et al, 2020[22]	Lumbo-pelvic sagittal alignment: poor in individuals over 50 years of age with severe KOA and has stronger relationship with progression severity of KOA in women than in men PT(°): (mean ± SD), KL1(15.8±7.5),KL2(20.1±8.8), KL3(21.4±9.2),KL4(24.7±9.5) (*p*=<0.01)	Not assessed	Not assessed

Abbreviations: *ASD* adjacent-segment disease, *β* beta coefficient, *BMI* body mass index, *CI* confidence interval, *DLS* degenerative lumbar spondylolisthesis, *DS* degenerative spodylolisthesis, *FI* femoral inclination, *K/*L Kellgren and Lawrence, *KFA* knee flexion angle, *KOA* Knee OA, *LBP* low back pain, *LL* lumbar lordosis, *M* male, MD mean difference, *NS* not specified, *OR* odds ratio, *OA* osteoarthritis, *PFA* pelvic femoral angle, *PI* Pelvic incidence, *PI-LL* pelvic incidence-lumbar lordosis, *PT* pelvic tilt, *RCT* randomized control study, *ROM* range  of motion, *SD* standard deviation, *SE* standard error, *SFA* sacrofemoral angle, *SS* sacral slope, *SVA* sagittal vertical axis, *SD* standard deviation, *TKA* total knee arthroplasty, *VAS* visual analogue scale, *WOMAC* Western Ontario and McMaster Universities Osteoarthritis Index

### Association between LBP and KOA

#### Biomechanical associations

##### Spinopelvic alignment

There were seven studies that have investigated biomechanical measures such as specific angles and alignment of the bones in relation to LBP and KOA in the literature. Development and progression of KOA in degenerative spondylolisthesis (DS) patients may be induced by significantly greater mismatches of lumbo-pelvic sagittal alignment [19]. Rate of double adjacent level of spondylolisthesis (condition in which a vertebral body shifts forward with an intact neural arch, compared to the vertebral body beneath it [24] in KOA group and non-KOA group) was 33.3% and 18.1%, respectively [19]. As a result of significantly greater PI (mean ± SD, 58.0° ± 10.4) and pelvic tilt (PT) (27.2° ± 9.8), double adjacent level spondylolisthesis with greater pelvic incidence-lumbar lordosis (PI-LL) (30.6 ± 10.0) is dominant in KOA patients with DS than patients without KOA. This shows that these factors are responsible in complicating KOA in patients with DS [19]. Elderly patients with degenerative lumbar spondylolisthesis (DLS) and severe KOA reported a different pelvic morphology than in patients with no to mild and moderate KOA [20]. They also presented with an increased sagittal malalignment and a lack of lumbar lordosis due to the double-level listhesis and greater knee flexion (mean ± SD, 10.1° ± 5.3) contracture. Parameters in lumbo-pelvic sagittal alignment is as follows: PT, LL, PI-LL, and SS of KOA group and non-KOA group were mean ± SD, 27.2° ± 9.8 and 22.2° ± 8.6, 40.4° ± 15.8 and 42.6° ± 14.3, 17.9° ± 15.1 and 10.3° ± 12.9, and 30.6° ± 10.0 and 30.6° ± 8.9, respectively. A greater pelvic retroversion (mean ± SD, 34.1° ± 10.8) may have activated in these patients as a compensatory mechanism [20]. Severe OA exhibited a significantly greater (mean ± SD, 56.7° ± 8.7) (*p* = 0.05) pelvic incidence (the angle between the line perpendicular to the sacral end plate at its midpoint and a line connecting this point to the axis of the femoral head), pelvic tilt, and knee flexion angle (KFA), along with a smaller degree (mean ± SD, 34.9° ± 14.6) of lumbar lordosis than in the mild-OA group (*p* = 0.26) [20].

Rate of radiographic adjacent-segment disease (ASD) (which is a condition with encompassing many complications of spinal fusion, including listhesis, instability, herniated nucleus pulposus, stenosis, hypertrophic facet arthritis, scoliosis, and vertebral compression fracture [25]) was observed to be higher in the severe OA group than in the mild OA group (38%) (*p* = 0.02) [20]. Patients with ASD in severe OA exhibited significantly greater PT (mean ± SD, 26.2° ± 7.0), along with less LL (38.7° ± 12.2), than the patients without ASD (*p* < 0.05). High PI (58.3°) is a risk factor for development of spondylolisthesis and KOA [16]. The incidence of knee OA was higher in individuals with a high PI (58.3°) compared to low PI (49.5°) (*p* = 0.03).

A significantly greater pelvic anterior tilt (44.68°) angle was found in the patients with KOA of both the LBP and non-LBP groups compared to healthy people without KOA or LBP [18]. There was no significant difference in anterior trunk inclination angle or sagittal alignment between KOA patients with and without LBP [9]. The sagittal alignment of spine-pelvis-lower extremity axis was significantly influenced by severe KOA [9]. The lumbar spine is served as the primary source of compensation, while hip flexion and pelvic anteversion increased for further compensation [9]. Changes in sagittal alignment may not be involved in the pathogenesis of LBP in this patient population [9]. Patients with severe KOA showed significant backward femoral inclination (FI), hip flexion, and forward spinal inclination (*p* < 0.001) compared with controls (without KOA) [9]. In addition, patients with FI of 10° showed reduced lumbar lordosis and significant forward spinal inclination compared with controls, whereas those with FI > 10° presented with significant pelvic anteversion and hip flexion [9]. Individuals over 50 years of age with severe KOA reported to have a poor lumbo-pelvic sagittal alignment [22]. Severity of KOA found to be related to lumbo-pelvic sagittal alignment; however, it does not relate to the global spinal balance [22]. KOA was found to be strongly related with the pelvic retroversion [22]. This pelvic retroversion may lead the progression of KOA [22]. Vice versa, knee joint degeneration may affect the pelvic retroversion [22]. According to kinematic chain reaction, pelvic retroversion is related to hip external rotation and varus knee deformity in standing position [22]. Varus knee alignment increases the medial tibiofemoral load and is associated with knee osteoarthritis [22]. Thus, sagittal lumbo-pelvic malalignment, especially pelvic retroversion, could lead to the progression of KOA. Furthermore, lumbar kyphosis in women was found to be associated with a lower Knee Society Knee Scoring System (KSS) symptom score [23].

##### Range of motion (ROM)

Both knee and spinal ROM were measured in relation to LBP and KOA. The knee flexion angle on the ipsilateral side bent to pick up was significantly smaller in both KOA groups (median with LBP — 9.11° and without LBP — 8.99°) than in the controls (median without KOA and LBP 15.45°) in the downward reach and pickup movements [18].

The patients with KOA in the LBP and non-LBP groups showed significantly smaller (median: with *LBP* = −27.65°; non-LBP = −27.44°) trunk flexion angles than that of the controls (without KOA or LBP, median = −40.43°), and the rotation angle of the non-LBP group was smaller (median = 6.01°) than that of the controls (9.15°) [18].

#### Clinical characteristics

Almost every clinical measure was worse among those who report back pain, including Health Assessment Questionnaire disability, pain, global severity, fatigue, and psychological status in people with KOA [6]. Pain and functional disability were commonly investigated in relation to LBP and KOA in the previous literature Table 3.

**Table 3:** Clinical causations/relationships between LBP and knee OA

Study	Clinical causations/relationships	
	Pain	Function/Disability
Chang et al, 2014 [17]	Diminished knee pain not associated with radiographic severity of lumbar spine degeneration (β= 0.08, *p*=0.28)	Diminished function: not associated with radiographic severity of lumbar spine degeneration (β= -0.02, *p*= 0.80)
	LBP severe grade (VAS 7–10): associated with knee pain (regression coefficient with 95% CI,-11.66 (-21.50 to -1.82))	Function: adversely affected by severe LBP (VAS score 7-10) (regression coefficient with 95% CI, -17.8 (-26.36 to -9.24))
		Poorer function: associated with moderate symptom grade (regression coefficient with 95% CI, -5.64 (-12.24 to 1.07)
		Diminished physical status: not significantly associated with radiographic severity of lumbar spine (β = -0.001, *p*=0.99)
		Physical status: affected by severe LBP (VAS score 7-10) (regression coefficient with 95% CI, -1.61 (-4.74 to -1.52)
Huang et al, 2014 [18]	Levels of back pain intensity component: higher inLBP group (median (IQR), with LBP 1.0 (1.0/2.0); non LBP 0.5 (0.0/1.0))	Physical disability: higher in LBP group (median (IQR), with LBP 9,(7.3/10.8); non LBP 3.5, (2.0/5.8)) Pain and functional disability of the patients with knee OA: no statistically significant difference between LBP and non LBP(median (IQR), with LBP 11.0 (9.3/15.0); 13.0 (10.0/14.0))
Iijima et al, 2018 [8]	LBP: (VAS score (mean ±SD), (3.53 ± 1.91)associated with increased disability level( β: 0.69; 95% CI: 0.01 to 1.37) (*p*=0.05)	Disability level: LBP is associated with increased disability level (*p* =0.05)
	Relationships of LBP and disability level: slightly increased in moderate to severe LBP ( β:1.01; 95% CI: 0.22 to 1.80) (*p*=0.01)	
	Relationship between knee pain intensity and disability level: higher in individuals with LBP ( β:0.62; 95%CI:0.51 to 0.73) than in those without LBP (β: 0.40; 95% CI: 0.32 to 0.49)	
Staibano et al, 2014 [21]	Back pain: none (55%) or very mild (28.9%) LBP (p <0.001) in preoperative TKA patients	Degree of disability due to back pain: minimal disability (mean±SD),(14.5 ±14) (due to back pain in preoperative TKA patients (p < 0.01) compared to moderate or severe (39.9 ±15.1) LBP patients
		Pain on the ODI: significantly higher among knee patients with a 68.4% (95% CI, 57.4–77.6%)
Stupar et al, 2010 [12]	Knee pain: not associated with LBP in individuals with KOA (*p* = 0.99)	Disability: not associated with LBP in individuals with KOA (β = 0; 95% CI, −3.39 to 3.39; *p* = 0.99)
Suri et al, 2010 [7]	Knee pain scores: substantially higher in patients with moderate and severe LBP (patients no. and percentage713 (89.6)) (*p*<0.001) and not associated with mild LBP (*p*=0.79)	LBP: significantly associated with increased functional score (β= 1; *p*<0.01)
		(WOMAC score with LBP β (SE) 6.5±4.1, without LBP 5.2±+ 3.4)
Taniguchi et al, 2021 [23]	LBP alone or LBP coexisting with lumbar kyphosis: was significantly associated with the knee pain and stiffness with lumbar kyphosis (mean± SD), (18.5 ±6.4) (*p* < 0.01) than without lumbar kyphosis(mean±SD), (20.0 ± 5.5) *p* < 0.01)	LBP and lumbar kyphosis: independently associated with a lower function (MD 95% CI, -4.96 (-7.56 to 13 -2.36) points for LBP alone, -4.47 (-8.51 to -0.43) points for lumbar kyphosis alone, and -13.86 14 (-18.86 to -8.86) points for the coexistence of LBP and lumbar kyphosis, respectively)
		Coexistence of LBP and lumbar kyphosis: was associated with a lower function in women (mean difference 95%CI, -4.49 (-6.42 to -2.55]) points)
Van Erp et al, 2020 [16]	Kneepain intensity: higher (*p*=0.37) in individuals with knee OA KL ≥ 2 and individuals with high PI (58.3) compared to low PI (49.5) (*p* = 0.05)	Not assessed
Wolfe et al, 1996 [6]	Back pain: strongly associated with knee pain (p=0.03)	Disability (*p*<0,01): strongly associated with back pain (*p*=0.03) Disability (1-1.9): OR 2.13, 95% CI (1.37, 3.30) Disability (≥2):: OR 6.85, 95% CI (2.87, 16.26)
	Knee pain: VAS (1-1.9): OR 2.18, 95% CI (2.03, 3.83)	
	VAS (≥2): OR 4.89, 95% CI (2.60, 9.20)	
Yasuda et al, 2020 [22]	Not assessed	Degree of disability due to back pain: Progression severity of KOA had more impact on stronger relationship with disability related LBP in (women > men) (*p*=0.02)ODI score:KL1(9.9±10.8), KL2( 12.2±11.9), Kl3(1±12.1), KL4 (16.1±13.0)
		ODI score: higher in the KL4 than in the KL1

Abbreviations: *ASD* adjacent-segment disease, *β* beta coefficient, *BMI* body mass index, *CI* confidence interval, *DLS* degenerative lumbar spondylolisthesis, *DS* degenerative spodylolisthesis, *HAQ* Health Assessment Questionnaire, *JKOM* Japanese Knee Osteoarthritis Measure, *K/L* Kellgren and Lawrence, *KOA* Knee OA, *KSS* Knee Scoring System, *LBP* low back pain, *M* male, *MCS* mental component summary, *MD* mean difference, *NS* not specified, *OR* odds ratio, *ODI* oswestry disability index, *OA* osteoarthritis, *PCS* physical component summary, *RDQ* Roland Morris Disability Questionnaire, *RCT* randomized control study, *SD* standard deviation, *SE* standard error, *SD* standard deviation, *SF 36* short-form 36, *TKA* total knee arthroplasty, *VAS* visual analogue scale, *WOMAC* Western Ontario and McMaster Universities Osteoarthritis Index

### Pain in concurrent LBP and KOA

Back pain is strongly associated with knee pain [6]. Compared to the primary KOA patients with mild LBP, patients with severe LBP had significantly poorer Western Ontario and McMaster Universities Osteoarthritis Index **(**WOMAC) pain score [17]. Roland-Morris Disability Questionnaire (RDQ) and Oswestry Disability Index (ODI) pain scores were higher (median = 9, non-LBP = 3.5) in the LBP group with KOA [18]. LBP was significantly associated with increased WOMAC knee pain score (*p* < 0.01) [7]. Although mild LBP was not associated with WOMAC knee pain score, moderate and severe LBP were each associated with substantially higher (95% *CI* = −11.6) WOMAC knee pain scores [17]. Knee pain intensity with LBP was higher (*β*: 0.62; 95% *CI*: 0.51 to 0.73) than in those without LBP (*β*: 0.40; 95% *CI*: 0.32 to 0.49) in individuals with LBP compared to no LBP in KOA [8]. There was another study to support no association between LBP and pain in KOA [12].

### Functional disability in concurrent LBP and KOA

LBP interacts with knee pain intensity and contributes to the disability level in individuals with KOA [8]. Coexisting LBP and knee pain had a stronger impact on disability level than in individuals with LBP than in those without LBP [8]. The presence of LBP was associated with increased disability level (with LBP (*β*: 0.62; 95% *CI*: 0.51 to 0.73) than in those without LBP (*β*: 0.40; 95% *CI*: 0.32 to 0.48)), and relationship between knee pain intensity and disability level was higher in individuals with LBP than in those without LBP [8]. More severe lumbar spine symptoms (visual analogue scale (VAS score ≥ 7)) were likely to adversely affect the WOMAC physical component summary and mental component summary scores of the SF-36 among patients with advanced KO [17]. In addition, a moderate lumbar spine symptom grade was associated with a poorer WOMAC function score [8].

ODQ and ODI scores were higher (11 and 9, respectively) in people with concurrent LBP and KOA [2, 8]. Moreover, the progression severity of KOA had more impact on stronger relationship with disability-related LBP in women than in men [22]. In women, the ODI score in people with severe OA was worse compared to that in mild OA [22].

Both LBP and lumbar kyphosis are useful clinical signals and indicate functional disability and knee symptoms in patients with knee OA [23]. LBP and lumbar kyphosis were independently associated with a lower KSS function score [23]. The coexistence of LBP and lumbar kyphosis in women was associated with a lower KSS symptom score [23].

There is evidence to support for having no clinical relationship between these two conditions. LBP was not associated with disability in individuals with KOA (*p* = 0.998) [12]. There was no statistically significant difference in the Lequesne’s index scores between the LBP and non-LBP groups with KOA [18]. ODI score and corresponding back pain disability among KOA patients indicated none or minimal disability in another study [21]. Patients with end-stage KOA were more likely to experience none or very mild LBP, with minimal disability due to back pain based on ODI score.

### Quality of the included studies

**Fig. 2:**
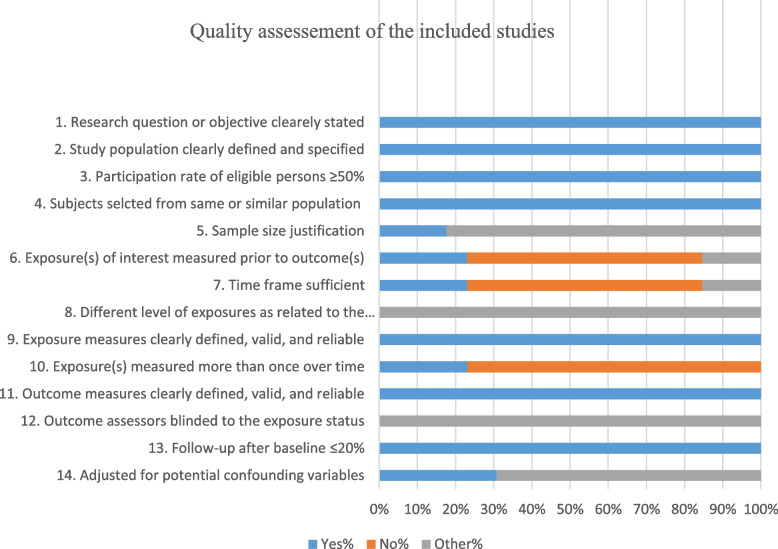
Quality assessment of the included studies

Figure 2 reveals the overall assessment of the quality of the included studies. Out of 14 items of the quality assessment, 6 criteria were adequately addressed by all the included studies. They have clearly described the objectives and study population and had a rate of ≥ 50% eligible persons, reliable and valid exposure measures and outcome measures, sufficient timeframe, and sufficient follow-up rate. However, sample size justification, power description, or variance and effect estimates should be clearly indicated in the methods (Fig. 2).

## Discussion

There were different biomechanical and clinical causations were revealed for the concurrent existence of KOA and LBP. Biomechanically, high pelvic incidence was found to be a risk factor for development of spondylolisthesis and KOA. Older people with degenerative lumbar spondylolisthesis and severe KOA reported a different pelvic morphology, increased sagittal malalignment with a lack of lumbar lordosis, and greater knee flexion contracture compared to no to mild and moderate KOA. Clinically, knee pain intensity was higher in KOA when presents with LBP. People with concurrent LBP and KOA have reported poor function with more disability. Despite research assessing the prevalence and clinical outcomes of cocurrent KOA and LBP, no attempts were made to pool the data about the mechanism or courses explaining the association between these two conditions in the literature [7, 8]. Furthermore, to our knowledge, no systematic reviews assessing the association between LBP and KOA have been found. Therefore, this study is the first to systematically explore this association, and several biomechanical and clinical causes were identified for this association.

Some biomechanical causations that exist between KOA and LBP were reported in this review [16, 19, 20]. It was found that high PI is a risk factor for development of spondylolisthesis and KOA [16]. Development and progression of KOA in patients with degenerative spondylolisthesis may be induced by significantly greater mismatches of lumbo-pelvic sagittal alignment [19]. Elderly patients with degenerative lumbar spondylolisthesis and severe KOA reported a different pelvic morphology with an increased (mean ± SD: *PI*, 56.7° ± 8.7 (*p* = 0.05); *PT*: 34.1° ± 10.8) sagittal malalignment with a lack of lumbar lordosis due to double-level listhesis and, a greater knee flexion (*p* = 0.02), in severe OA (mean ± SD: 10.1° ± 5.3) group than the mild-OA (4.9° ± 6.8) contracture than in patients with no to mild and moderate KOA [20].

Biomechanical relationship between LBP and KOA suggestively can be due to an altered compensation mechanism in lower limb joints and musculature. The normal upright standing posture of the body is maintained by correct alignment of the spine, the pelvis, the lower extremities (LEs), and associated musculature attached to those structures [11]. Impairment of bony structures and weakness or imbalance of these structures may cause disorders of the lower limb or vice versa [11]. Decreased knee flexion and lumbar lordosis and increased sacropelvic angle cause sacroiliac joint problems resulting in LBP [26]. Furthermore, this phenomenon is caused by changes in the spinal alignment (lumbar kyphosis) and knee flexion position, referred to as knee-spine syndrome. In the sagittal plane, spinal kyphosis increases pelvic retroversion, hip extension, knee flexion, and ankle dorsiflexion as compensation. These compensatory mechanisms induce load on the knee joint, resulting in the progression of KOA. Severe KOA influences on sagittal alignment of the spine-pelvis and LE axis [9]. Vice versa, degenerative changes in lumbar spine and loss of lumbar lordosis may be associated with degenerative changes in the knee [10]. Limitation of knee extension significantly increases with reduced lumbar lordosis [10]. Furthermore, there is a correlation between endurance of muscles around the lumbar area and balance in people with KOA [27]. Poor dynamic balance was reported in individuals with weak core muscles endurance [27].

In terms of clinical relationships, there was a relationship observed in pain and disability between KOA and LBP. Knee pain intensity was higher in KOA when it is presented with LBP [8]. People with concurrent LBP and KOA have reported overall poor body function with more disability [8, 22]. Incorrect alignment, and stressed structures because of the malalignment, may cause the pain aggravations [28]. The subchondral trabecular bone microarchitecture is associated with the hip-knee-ankle angle and OA severity [28]. With the increase of the knee alignment deviation and OA severity, the subchondral trabecular bone of the affected side tibial plateau increased in bone volume, trabecular number, and trabecular thickness and decreased in trabecular separation [28]. LBP and lumbar kyphosis are both useful clinical indicators of functional disability and knee symptoms in patients with knee OA [23]. Coordination of the alignment of the spine, pelvis, and lower extremity maintains a stable and ergonomic upright standing position, particularly in the sagittal plane [8, 29]. Pathology in any segment of the trunk or lower leg can interrupt the overall postural equilibrium, resulting in compensatory changes in other segments. Postural abnormalities may play a role in the occurrence of LBP by creating concentrations of stress [30, 31]. This may alter weight-bearing axis of lower limb resulting increasing degenerative changes in the knee joint [28]. Degenerative changes in the knee may be provocative of knee pain. Low back pain is biomechanically linked to knee pain via the so-called knee-spine syndrome [10]. These symptoms may eventually lead to global disability if left untreated or not appropriately managed.

The findings of this systematic review are clinically important and relevant because they were associated with higher co-occurrence [17], pain intensity [8], disability [8], and fall’s risk [32]. Hence, it is important to assess core strength, back stiffness, or any signs of back pain as a preventive strategy. Early assessment and screening for LBP in KOA, and early core strengthening, would immensely help to deteriorate the progression of KOA into a knee-spine syndrome which may lead to LBP [8, 10, 33]. In the presence of co-occurrence, it is vital that the clinician considers treating both the conditions rather than single KOA.

In terms of the quality assessment of the current review, all studies have clearly described the research objectives indicating a characteristic of a higher quality scientific research. All studies have described their study population, and this helps in generalizing the findings. In every study, more than 50% of eligible persons participated in the study, and this reflects an adequate representation of the target population. More than half of the criteria were adequately addressed, and this increases the internal and external validity of the studies. However, sample size justifications should be clearly indicated in future research.

There were several limitations of the present review. We have limited the search strategy to studies only written in English. We considered only studies assessing people with KOA; however, we excluded people with knee pain. Therefore, further studies are warranted to explore any association between LBP and knee pain. A systematic search on efficacy of concurrent treatment for LBP in KOA is required. Future research should focus on assessing the effect of early core strengthening along with lower limb strengthening for KOA and LBP. Future studies should be enhanced with a proper sample size justification, a power description, or variance and effect estimations.

## Conclusion

The present review reported several biomechanical and clinical causations for the concurrent existence of KOA and LBP. Early comprehensive assessment is required in managing KOA and LBP. Further trials with high-quality methodology are warranted to assess the effects of the exercise programs consisting of both lumbar and knee exercises for KOA patients.

## Supplementary Information

**Additional file 1.** PRISMA_2020_checklist.

**Additional file 2.** Search strategy.

## Data Availability

The datasets generated and/or analyzed during the current systematic review are available from the corresponding author upon a reasonable request.

## Abbreviations

LBP: Lower back pain
OA: Osteoarthritis
KOA: Knee osteoarthritis
KSS: Knee Society Knee Scoring System
PI: Pelvic incidence
OA: Osteoarthritis
QOL: Quality of life
LEs: Lower extremities
LE: Lower extremity
ROM: Range of motion
DS: Degenerative spondylolisthesis
LL: Lumbar lordosis
PI-LL: Pelvic incidence-lumbar lordosis
PT: Pelvic tilt
DLS: Degenerative lumbar spondylolisthesis
KFA: Knee flexion angle
ASD: Adjacent-segment disease
FI: Femoral inclination
WOMAC: Western Ontario and McMaster Universities Osteoarthritis Index
RDQ: Roland-Morris Disability Questionnaire
ODI: Oswestry Disability Index
VAS: Visual analogue scale
TKA: Total knee arthroplasty
SS: Sacral slope
