# Supplementary material for: Biomechanical and clinical relationships between lower back pain and knee osteoarthritis: a systematic review
Source: Syst Rev. 2023 Mar 2;12:28. doi: 10.1186/s13643-022-02164-3 (PMC9979420; doi:10.1186/s13643-022-02164-3)
Supplement: Supplementary file 2 — Additional file 2. Search strategy. [file 13643_2022_2164_MOESM2_ESM.docx]

MEDLINE VIA OVID

| 1 | Low back pain.mp. or Low Back Pain/ or Back Pain/ or back pain.mp. or Spondylolisthesis/ or spondylolisthesis.mp. or backache.mp. or back-ache.mp. or Lumbago.mp. or dorsalgia.mp. or (lumbar adj pain).ti,ab. or coccydynia.ti,ab. or sciatica/ or sciatica.ti,ab. or Intervertebral Disc Displacement/ or Spondylosis/ or spondylosis.mp. or spondylitis/ or spondylitis.mp. or ischalgia.mp. or discitis.mp. or Discitis/ or arachnoiditis.mp. or Arachnoiditis/ or (slipped adj (disc* or disk*)).mp. or (degenerat* adj (disc* or disk*)).mp. or (herniat* adj (disc* or disk*)).Emp. or (displace* adj (disc* or disk*)).mp. or (prolap* adj (disc* or disk*)).mp. or (back adj2 pain).mp. or back disorder*.mp. |
| --- | --- |
| 2 | Osteoarthritis/ or osteoarthr$.mp. or (degenerative adj2 (joint or arthriti$)).mp. or Osteoarthritides.mp. or Arthritis, Degenerative.mp. or (Degenerative adj2 Arthritis).mp. or Osteoarthrosis Deformans.mp. or (osteoarthriti* adj2 knee*).mp. or (arthritis adj noninflammatory).mp. or (arthrosis or arthroses).mp. |
| 3 | 1 and 2 |
| 4 | limit 3 to (humans and ("all adult (19 plus years)" or "young adult (19 to 24 years)" or "adult (19 to 44 years)" or "young adult and adult (19-24 and 19-44)" or "middle age (45 to 64 years)" or "middle aged (45 plus years)" or "all aged (65 and over)" or "aged (80 and over)")) |

EMBASE VIA OVID

Same above, except age limitation:

4 limit 3 to (human and (adult <18 to 64 years> or aged <65+ years>))

SCOPUS

(TITLE-ABS-KEY("back pain*" OR backache OR lumbago OR dorsalgia OR coccydynia OR sciatica OR spondylosis OR spondylitis OR ischialgia OR discitis OR arachnoiditis)) AND (TITLE-ABS-KEY(osteoarthriti* OR osteoarthritis OR "osteoarthrosis deformans" OR arthrosis OR arthroses OR "knee osteoarthriti*"))
